# Visual and psychological morbidity among patients with autosomal dominant optic atrophy

**DOI:** 10.1111/aos.12077

**Published:** 2013-03-04

**Authors:** Maura Bailie, Marcela Votruba, Philip G Griffiths, Patrick F Chinnery, Patrick Yu-Wai-Man

**Affiliations:** 1 Department of Ophthalmology, Royal Victoria InfirmaryNewcastle upon Tyne, UK; 2 School of Optometry and Vision Sciences, Cardiff UniversityCardiff, UK; 3 Cardiff Eye Unit, University Hospital of WalesCardiff, UK; 4 Department of Neurology, Royal Victoria InfirmaryNewcastle upon Tyne, UK; 5 Wellcome Trust Centre for Mitochondrial Research, Institute of Genetic Medicine, Newcastle UniversityNewcastle upon Tyne, UK

Editor,

Autosomal dominant optic atrophy (DOA) is the most common inherited optic nerve disorder seen in neuro-ophthalmological practice (Yu-Wai-Man et al. 2011). The majority of patients with DOA harbour pathogenic mutations within the *OPA1* gene, which codes for an inner mitochondrial membrane protein intricately involved in mitochondrial biogenesis, mitochondrial DNA replication and network stability (Amati-Bonneau et al. 2009). The pathological hallmark of this nuclear mitochondrial disorder is progressive retinal ganglion cell loss, leading to optic nerve degeneration and bilateral visual failure from early childhood. About 20% of *OPA1* mutation carriers will develop a more severe form of the disease (DOA+) characterized by prominent extraocular neurological features including deafness, ataxia, peripheral neuropathy and myopathy (Yu-Wai-Man et al. 2010). The visual and psychological impact of DOA has not yet been reported. The aim of this study was to quantify the functional impact of DOA on patients’ quality of life and to determine, in particular, whether the additional neurological burden that develops in DOA+ further compounds the visual disability in this group of patients.

A total of 38 patients harbouring confirmed pathogenic *OPA1* mutations were recruited into this study: 30 patients with isolated optic atrophy and eight patients with DOA+ phenotypes. A telephone interview was conducted by a single investigator (MB), who was blinded to the patients’ mutational status and clinical history, using two well-validated questionnaires: the Visual Function Index (VF-14) (Kirkman et al. 2009) and the Hospital Anxiety and Depression Scale (HADS) (Hinz et al. 2010). Visual acuity data were obtained from the patients’ records. This study had the relevant institutional ethical approval and complied with the Declaration of Helsinki.

The mean VF-14 score for the entire patient cohort was 37.3 (Standard deviation = 25.6). Patients with DOA+ had significantly worse visual acuities and VF-14 scores compared with those with pure DOA (Fig. 1A). Borderline or definite symptoms of anxiety and depression were present in 19/38 (50.0%) and 7/38 (18.4%) patients, respectively. Compared with the general adult population, the mean HADS scores were significantly increased for both anxiety (p = 0.0490) and depression scales (p = 0.0287) (data not shown). On subgroup analysis, significantly higher depression scores, but not anxiety scores, were found in patients with DOA+ compared with those with pure DOA (Fig. 1B,C). There was a statistically significant correlation between VF-14 score and (i) LogMAR vision, (ii) anxiety score and (iii) depression score (Fig. 1D–F).

**Fig 1:**
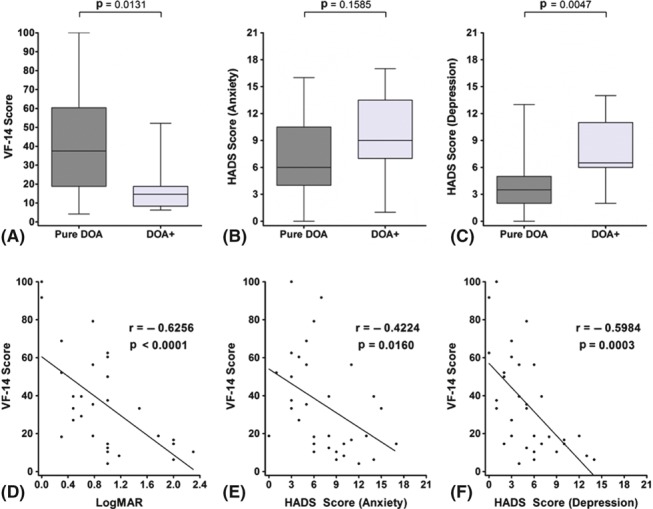
*OPA1* patient cohort analysis. The data have been represented as box plots with the whiskers representing the minimum and maximum values. The ends of the boxes are the upper and lower quartiles, the vertical lengths of the boxes indicate the interquartile range, and the lines within the boxes represent the median values for each group. Group comparisons were carried out with the unpaired *t*-test using GraphPad™ Prism v5 statistical software (San Diego, CA, USA). LogMAR = logarithm of the minimum angle of resolution. (A) VF-14 score (pure dominant optic atrophy (DOA) group: mean = 42.40, standard deviation (SD) = 25.51; DOA+ group: mean = 17.74, SD = 14.73); (B) Anxiety score (pure DOA group: mean = 7.20, SD = 4.02; DOA+ group: mean = 9.63, SD = 5.01); (C) Depression score (pure DOA group: mean = 4.03, SD = 3.02; DOA+ group: mean = 7.88, SD = 3.87); (D) Correlation between VF-14 score and LogMAR vision (*r * =  Spearman rank correlation coefficient); (E) Correlation between VF-14 score and anxiety score; (F) Correlation between VF-14 score and depression score.

Patients with DOA experience significant difficulties in their activities of daily living, the severity being comparable to the degree of functional handicap in Leber hereditary optic neuropathy (LHON) – a classical primary mitochondrial DNA disorder that typically presents with catastrophic bilateral blindness in early adulthood (Kirkman et al. 2009). Unlike LHON, visual failure in DOA has a more insidious course, but it is invariably progressive, and the VF-14 score clearly indicates the considerable visual morbidity associated with this disorder. Furthermore, although half of all *OPA1* mutation carriers will eventually fulfil the legal requirement for blind registration (LogMAR < 1.30 in the United Kingdom), impaired activities of daily living were also apparent for those with visual acuities below this threshold, and this patient group should not be denied support from social services. Another observation from our study is the significant psychological impact of DOA, with levels of anxiety and depression approaching those seen in patients undergoing cancer treatment (Hinz et al. 2010). Importantly, the psychological distress seemed magnified in patients manifesting DOA+ phenotypes. From a practical perspective, these patients therefore represent a high-risk group that requires greater clinical input and improved access to rehabilitative services to lessen the impact of the neurological complications on the already considerable visual deficits.

## Contributorship Statement

All authors contributed to this article through each of the following: (1) conception and design, analysis and interpretation of data; (2) drafting the article and revising it critically for important intellectual content; and (3) giving final approval for the version to be published.

## Disclosures

All the listed authors in this manuscript report no relevant financial disclosures or conflicts of interest.
